# Cylindrospermopsin Biodegradation Abilities of *Aeromonas* sp. Isolated from Rusałka Lake

**DOI:** 10.3390/toxins8030055

**Published:** 2016-02-25

**Authors:** Dariusz Dziga, Mikolaj Kokocinski, Anna Maksylewicz, Urszula Czaja-Prokop, Jakub Barylski

**Affiliations:** 1 Department of Plant Physiology and Development, Faculty of Biochemistry, Biophysics and Biotechnology, Jagiellonian University, Gronostajowa 7, 30387 Krakow, Poland; anna.maksylewicz@uj.edu.pl (A.M.); ula.czaja-prokop@uj.edu.pl (U.C.-P.); 2 Department of Hydrobiology, Faculty of Biology, Adam Mickiewicz University, Umultowska 89, 61-614 Poznań, Poland; kok@amu.edu.pl; 3 Department of Molecular Virology, Faculty of Biology, Adam Mickiewicz University, Umultowska 89, 61-614 Poznań, Poland; erytropoeta@gmail.com

**Keywords:** cylindrospermopsin, biodegradation, *Aeromonas* sp., Polish lakes

## Abstract

The occurrence of the cyanobacterial toxin cylindrospermopsin (CYN) in freshwater reservoirs is a common phenomenon. However, the biodegradation of this toxin in environmental samples has been observed only occasionally. In this work the biodegradation ability of cylindrospermopsin was investigated based on isolates from lakes with previous cyanotoxin history. Bacterial strains were identified based on the 16S rDNA and rpoD gene comparison. CYN biodegradation was monitored using the HPLC method. The R6 strain identified as *Aeromonas* sp. was documented as being capable of CYN removal. This biodegradation was dependent on the pH and temperature. Additionally, the stimulation of the growth of the R6 strain in the presence of CYN was indicated. Our discovery supports the hypothesis that (in analogy to the well-known phenomenon of microcystin biodegradation) in lakes dominated by potential CYN-producing cyanobacteria, the processes of microbial utilization of this toxin may occur.

## 1. Introduction

Increased eutrophication of surface water may result in toxic cyanobacterial blooms and the presence of secondary toxic metabolites. Among them, cylindrospermopsin (CYN) constitutes a relatively new problem and its occurrence in temperate climates is increasing. CYN-producing strains e.g. *Cylindrospermopsis raciborskii* or *Aphanizomenon ovalisporum*, originally thought to proliferate only in tropical regions of the world, are now being detected in more temperate climates, suggesting that these potentially toxin-producing cyanobacteria are highly adaptive, which has important implications for water authorities worldwide. In addition, climate change also creates more favorable conditions for these cyanobacteria to proliferate in water supplies [1]. Currently, in some European reservoirs, CYN may even be the predominant freshwater cyanotoxin. Apart from Germany, Spain, Finland, and Italy, this toxin was also detected in other locations in Europe, including Poland, where it was first found in 2006 in two shallow eutrophic lakes [2]. Recent studies revealed frequent occurrence of CYN in the lakes of western Poland, where this toxin was detected in 13 out of 36 investigated lakes [3]. CYN findings in European fresh waters were reported with maximum concentrations in the range of 9–126 µg·L^−1^ [4,5]. Therefore, it is likely that this toxin is commonly found in European waters and should be considered a serious threat to human health and to wildlife.

CYNs are relatively stable and resistant to chemical hydrolysis or oxidation. Their persistence and detoxification in the aquatic environment are important problems for public health. CYN is a highly biologically active alkaloid, interfering with several metabolic pathways. It inhibits protein synthesis, has hepatotoxic, general cytotoxic, genotoxic, and neurotoxic effects and is considered a potential carcinogen [6]. In mammals, cylindrospermopsin intoxication involves damage to multiple organ systems (liver, kidney, thymus, and heart). In humans CYN poisoning may correlate with a higher incidence of intestinal tract cancers [7]. Additionally, this toxin may be transferred to humans following its accumulation in aquatic organisms eaten as food [8].

A large fraction of CYN (68%–98%) occurs extracellularly [5,9], which may have important implications if concentrations in waters are in a health-relevant range (>1 µg·L^−1^ for drinking water [10]). The ambient concentration of cyanotoxins is a function of several different factors such as adsorption to particles, thermal decomposition aided by pH, accumulation into aquatic plants and animals, photolysis, and biodegradation by natural aquatic organisms [11]. The fate of CYN, similarly to other cyanotoxins may depend on biodegradation in water, as well as in sediment [12], and it could be the main pathway for toxin elimination. Furthermore, there is no or only little CYN sorption in sandy and silty sediments [13]. However, to date little has been published with respect to the biodegradation of CYN. Chiswell [9] and Senogles *et al.* [14] suggested that biodegradation was an important process in the removal of CYN from water bodies once the toxin was released from cyanobacterial cells. Smith *et al.* [15] showed that biodegradation of CYN occurred in many different environments that were contaminated with toxic *C. raciborskii* cells and lag periods were initially observed prior to the commencement of CYN biodegradation. In contrast, a work of Wormer *et al.* [16] showed no degradation of CYN by bacteria during a 40-day study. However, little is known about specific strains that may be involved in CYN utilization [17]. Additionally, some strains of probiotic bacteria have been proved to be efficient in the removal of several different cyanobacterial toxins, including CYN [18].

The objectives of this study were: (1) to select and identify CYN biodegraders among bacterial strains occurring in the lakes with potential CYN producers; (2) to examine the influence of pH and temperature on biodegradation of CYN by identified bacterial strain with CYN degradation capability; and (3) to investigate the influence of CYN on the growth of the CYN biodegrader.

## 2. Results

### 2.1. Activity of Isolated Strains towards CYN and Identification of CYN Biodegraders

The limnological characteristics of the investigated lakes (provided in Table S1) indicated that these reservoirs were highly eutrophic. Previous studies showed abundant occurrence of cyanobacteria in these lakes including *Cylindrospermopsis raciborskii, Aphanizomenon flos-aquae, Aphanizomenon gracile*, and *Chrysosporum bergii*, all considered as potential producers of CYN [19,20]. The present study confirmed the occurrence of these species with *A. gracile* being the most abundant. Recently CYN was detected in Lake Kierskie [21]. CYN occurrence in Lake Rusałka has not been analyzed to date but, due to a similar phytoplankton community, its production by dominant cyanobacterial strains is possible. Altogether, 16 bacterial strains were isolated and tested for CYN biodegradation. Among these strains, only one R6 strain collected from Lake Rusałka exhibited the ability to degrade CYN. During a six-day incubation of the toxin with the R6 strain bacterial cells, about 25% reduction of CYN concentration was observed in comparison with the control (3.00 ± 0.11, 2.77 ± 0.08, 2.49 ± 0.13, 2.24 ± 0.02 µg·L^−1^ of CYN at 0, 1, 3, and 6 days of incubation with the R6 cells, respectively).

**Figure 1:**
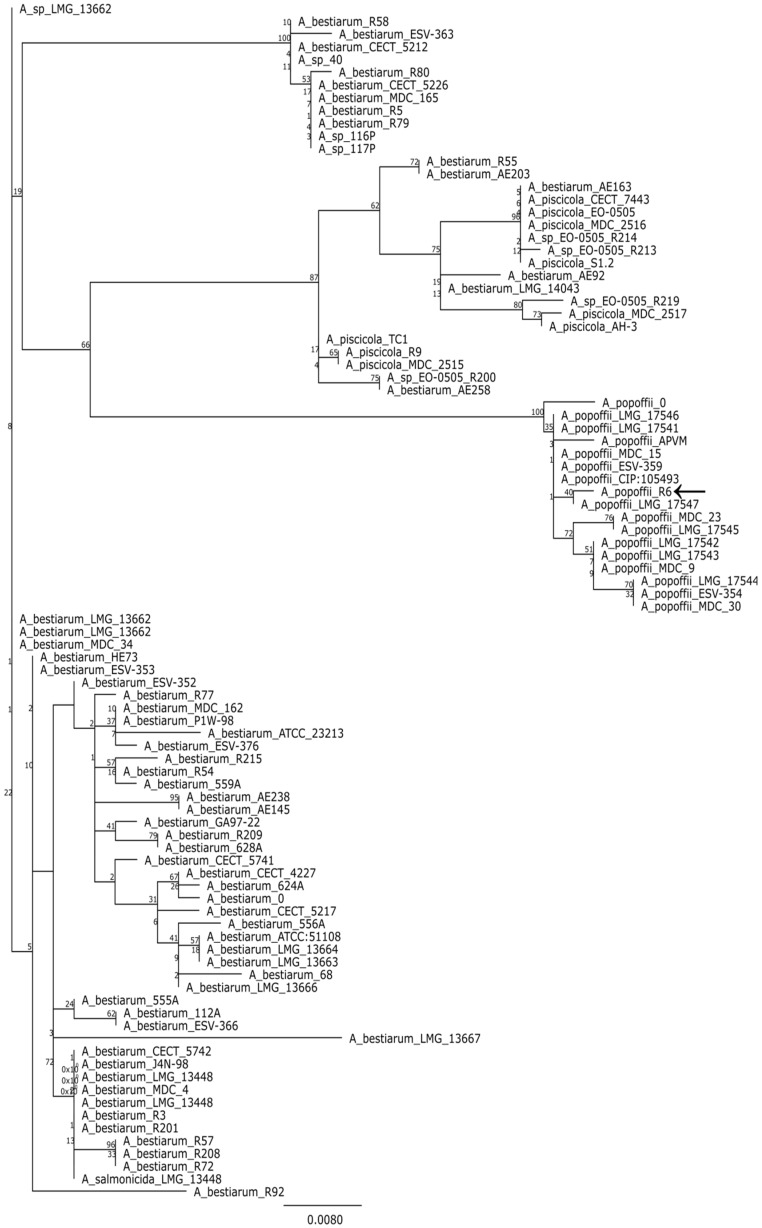
Majority consensus maximum likelihood tree (100 bootstrap replicates) based on a comparison of rpoD genes. Branch labels indicate bootstrap support; leaf names correspond to strain designations. The analyzed sequences are listed in the Supplementary materials.

MegaBLAST analysis of partial 16S rDNA sequence (Table S2) revealed that strain R6 is a member of the genus *Aeromonas*. Since variability of 16S region is usually insufficient to delimit the species within this genus [22], we analyzed the gene encoding the RNA polymerase sigma factor (rpoD) instead. To put our strain in phylogenetic context, we used BLAST to retrieve the 100 most similar sequences from the genbank and compared the rpoD gene to them. The resulting alignment was used to make a maximum likelihood tree. An analysis of the tree suggest that R6 may be a strain of *Aeromonas popoffii* but only a point mutation distinguishes the studied sequence from *A. bestiarum* (Figure 1).

### 2.2. Biodegradation of CYN at Different Temperatures and pH

**Figure 2:**
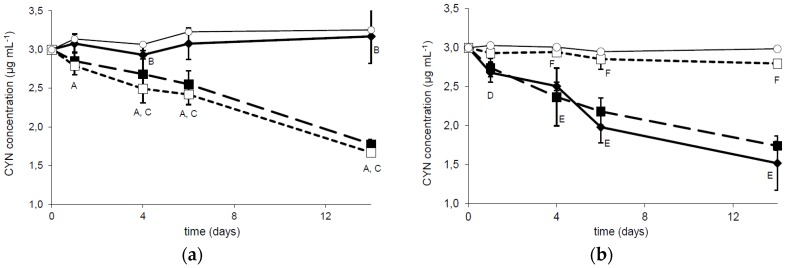
Dependence of CYN biodegradation by the R6 strain on the temperature (**a**) and pH (**b**). The solid, dashed, and dotted lines correspond to the column temperature 4 °C, 20 °C, and 30 °C (**a**) and to pH 6.5, 8.0, and 9.5 (**b**), respectively. Controls are indicated by solid lines with white circles. Errors indicate standard deviation (*n* = 3). Statistically significant differences: A—control *versus* 20 °C and 30 °C at 1, 4, 6, and 14 days; B—between control and 4 °C at 4 and 16 days; C—4 °C *versus* 20 °C and 30 °C at 4, 6, and 14 days; D—after 1 day between control and 6.5 pH; E—at 4, 6, and 14 days control *versus* 6.5 and 8.0 pH; F—9.5 pH *versus* 6.5 and 8.0 pH. See more details in Table S3.

Bacterial strain R6 has been investigated to examine the influence of temperature and pH on the biodegradation capability. A comparison of CYN concentration during two weeks of incubation with R6 strain at three different temperatures indicated that the tested bacterial strain expressed similar activity against the toxin at 20 and 30 °C (Figure 2a). The CYN removal was 47% and 49%, respectively, whereas at 4 °C the biodegradation was minimal (only 7% CYN was removed). The observed reduction in CYN concentration was relatively stable during the experiment, with a slightly faster rate during the first day of incubation. A similar biodegradation activity of R6 strain was observed at pH 6.5 and 8.0 (48.9% and 41.5% reduction of CYN concentration, respectively). At pH 9.5 only 6.5% of CYN was degraded in comparison with the control (Figure 2b).

**Figure 3:**
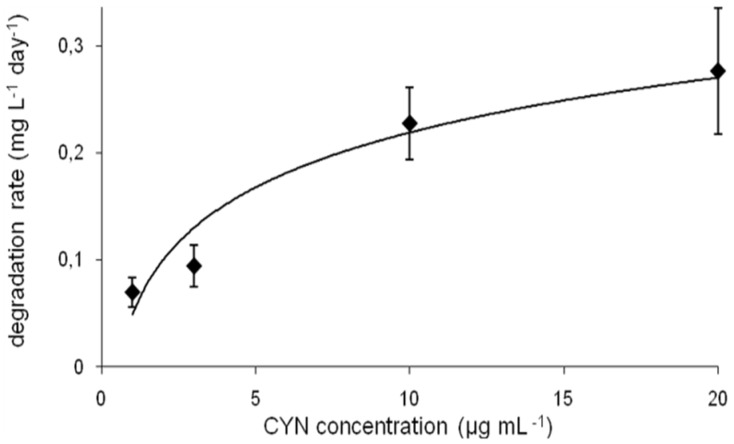
CYN biodegradation rate at different initial concentrations of toxin (errors indicate standard deviation (*n* = 3).

The calculated degradation rate measured at different CYN initial concentrations varied between 0.7 and 0.28 mg·L^−1^·d^−1^ (Figure 3). However, this parameter changed faster in the range 1–10 µg·mL^−1^ of CYN (by factor 3.3), whereas at higher CYN concentration range (between 10 and 20 µg·mL^−1^) this factor was 1.2. The degradation rate was dependent on the pH and the temperature (Figure S1).

### 2.3. Growth of the R6 Strain in the Presence of CYN

**Figure 4:**
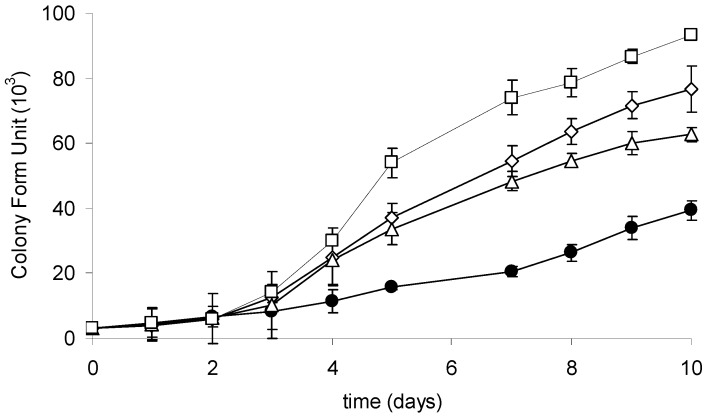
Growth curves of the R6 strain in the control culture (black circles) and at different initial CYN concentrations: 3 µg·mL^−1^ (white triangles), 10 µg·mL^−1^ (white rhombus), and 20 µg·mL^−1^ (white squares). Controls are indicated by solid lines with white circles. Errors indicate standard deviation (n = 3). Statistically significant differences: in three-day control *versus* 10 and 20 µg·mL^−1^; in five-day control *versus* 3, 10, and 20 µg·mL^−1^; in 10-day between all experimental groups. See more details in Table S3.

The growth of the investigated strain culture was monitored during a 10-day cultivation with or without CYN supplementation. At the end of the experiment, a significant upregulation of the growth of R6 strain exposed to CYN was observed (Figure 4). The number of cells was significantly higher at three (3, 10, or 20 µg·mL^−1^) tested CYN concentrations in comparison with the control. The generation time of cells exposed to the highest applied CYN concentration (20 µg·mL^−1^) was 1.5 times higher than in the control. Additionally, a lag phase in response to CYN has been indicated; a higher (compared to the control) growth rate of cells exposed to CYN was observed in the third day of cultivation.

## 3. Discussion

The known cyanotoxin biodegraders are usually found in water reservoirs with previous cyanobacterial history. Most probably bacterial communities from such lakes can use toxins along with other organic compounds as a source of carbon and energy. For this reason, screening for natural CYN biodegraders should be performed using the samples collected from the lakes with recent CYN producer history. In this work, *Aeromonas* sp. strains as potential CYN degraders were isolated from both investigated lakes. However, only one strain R6 from Lake Rusałka showed CYN degradation capability, indicating occurrence of a mixture of CYN-degrading and CYN-non-degrading strains within the bacterial community. In Lake Rusałka CYN occurrence has never been analyzed but the phytoplankton community was similar to Lake Kierskie, including the same CYN potential producers documented in previous reports [19,20]. It is interesting to remark that the strain of *Aeromonas veronii* v-s-03 was recently reported to be capable of MC degradation [23]. Unfortunately, since the 16S sequences used by these authors are remarkably similar among many *Aeromonas* species, the exact phylogenetic relation between v-s-03 and the R6 remains unknown.

The influence of different environmental conditions such as temperature or pH on the cyanotoxin (bio)degradation processes is commonly investigated. Degradation of CYN in sediments [12,13] was studied at alkaline conditions (pH 8). Observation of the CYN degradation in a water column [9] indicated that variation in water pH did not affect this process. On the other hand, biodegradation of CYN observed in the presence of a defined microorganism is rather pH dependent. In this paper we documented that the R6 strain degrades CYN more effectively between pH 6.5 and 8.0, whereas more alkali conditions (pH 9) caused an almost complete reduction of its activity toward CYN (Figure 2b and Figure S1), which may be caused by lower viability in such conditions. Similar effects were indicated by Mohamed *et al.* [17], who reported that the highest biodegradation rates (by *Bacillus* AMRI-03 strain) were at neutral and slightly alkaline conditions (pH 7–8). Chiswell *et al*. [9] reported that slow CYN decomposition in water occurs between 4 and 50 °C. Furthermore, several studies documented that temperature close to 20 °C is optimal for CYN biodegradation in the natural environment. For example, Smith *et al.* [15] documented that the highest rate of CYN decomposition was at 25 °C, whereas at 35 °C this process was slower. Similarly, temperature plays an important role in biological decomposition of the investigated toxin in the presence of selected bacterial strains. As was indicated by Mohamed *et al.* [17] and in the present study, the optimal conditions ranged between 20 and 30 °C (Figure 2a and Figure 3c). Such a profile of biodegradation is important due to an increased presence of CYN in temperate climates. The biodegradation rate measured at different initial toxin concentrations allows us to predict the potential efficiency of decomposition of the investigated strains. However, to date there has been no data documenting the biodegradation rate of CYN, so our data cannot be compared. On the other hand, we have relatively broad knowledge about the efficiency of MC degraders. For example, the calculated MC degradation rates of *Sphingomonas* sp. are very different and range between 0.12 and 13 mg·L^−1^·day^−1^ [24]. For the R6 strain (Figure 3), this parameter was 0.23 and 0.28 (10 and 20 µg·mL^−1^ of initial CYN concentration, respectively). This suggests that the R6 strain is rather less effective in cyanotoxin utilization than most known MC degraders; however, more comprehensive analyses are necessary to describe the actual efficiency of different bacterial species in cyanotoxin degradation.

We showed that CYN presence stimulates the growth of bacteria capable of its utilization (Figure 4). Cyanobacterial strains coexist naturally with certain species of bacteria that may use secondary metabolites (including toxins) as an additional source of organic compounds. We postulate that CYN may be used as an additional C and N source and may thus enhance the growth of bacteria utilizing the toxin. Furthermore, the species that coexist with CYN-producing cyanobacterial strains, e.g., belonging to the *Aphanizomenon* genera, should be of particular interest due to their potential ability to degrade CYN. Bacterial strains belonging to *Variovorax* sp., isolated in the present study from the *Aphanizomenon gracile* culture, did not express CYN degradation activity (data not shown). However, such heterotrophic microorganisms should be explored more comprehensively. Similarly, due to the observed CYN degradation in sediments [12], such an environment is also an alternative source of microorganisms with CYN degradation capability.

The mechanism of CYN degradation by the R6 strain remains unknown and it is a challenge to learn about both the products of CYN biodegradation and the enzymes involved in this process. It may be assumed that CYN is also utilized by bacteria as an organic substrate and that such microbial strains have a specified enzymatic pathway for this process. Similarly to MC biotransformation [24], enzymatic degradation of CYN probably requires both biochemical pathways of such substrate utilization and the genetic machinery to express the proteins involved in this process. In analogy to the research that allowed us to document the enzymes and genes involved in the hydrolysis of microcystin, a similar approach may be employed to investigate the mechanism of CYN degradation.

## 4. Materials and Methods

### 4.1. Sample Collection and Separation of Bacterial Strains

Water samples were collected in August 2014 from the surface layer (0–0.5m) in the center of two lakes, Rusałka and Kierskie Małe, located in the Wielkopolska region of western Poland. Their visibility was measured on sampling with a Secchi disk. Samples, collected in sterile 50-mL plastic flacons, were delivered immediately after sampling to the laboratory. Chlorophyll concentration was determined in the laboratory by the acetone method, as previously described [21]. Each sample was serially diluted and plated on Water Plate Count Agar. The bacteria were cultured at 20 °C until countable colonies appeared. Individual colonies were streaked to pure culture and stored at 4 °C for further analyses. Additionally, phytoplankton samples from the same sampling station were collected using a 0.5-m “Limnos” sampler. CYN concentration in Lake Kierskie Małe was determined using the HPLC-MS/MS method of an earlier study [21].

### 4.2. Phytoplankton Analysis

The phytoplankton samples were preserved with an acid Lugol solution immediately after sampling and were stored in cool and dark conditions before being analyzed. The phytoplankton were counted using a Fuchs-Rosenthal chamber (Carl Roth GmbH, Karlsruhe, Germany). Phytoplankton identification and counts were conducted using a Carl Zeiss light microscope (Axioskop 2 Mot, Württemberg, Germany) under 400× *g* magnification. At least 400 specimens were counted to reduce the error to <10%.

### 4.3. Identification of the Bacteria

Each of the 16 tested strains was derived from a single colony on the original plate. Cells from a single colony were lysed for 10 min at 95 °C with 0.5% IGEPAL CA-630 (Sigma-Aldrich, St. Louis, MO, USA) in 10 mM Tris-HCl buffer pH 8.5. After rapid cooling to 4 °C, cell debris was removed by centrifugation and supernatant was used as a template for amplification of 16S rDNA and rpoD genes (for cycling conditions and primers see Table S2). The PCR products were sequenced with a 3130× Genetic Analyzer (Applied Biosystems, Thermo Fisher Scientific, Waltham, MA, USA). To provide phylogenetic background for the analyzed strain, the obtained sequences were compared with those available in the GenBank database using a megaBLAST tool (National Center for Biotechnology Information, Bethesda, MD, USA) [25]. The top 100 hits for the rpoD sequence were retrieved from the database for further analysis. After the removal of redundant records, the sequences were aligned with MAFFT (v7.017, G-INS-I algorithm, Computational Biology Research Center, Tokyo, Japan) and a maximum likelihood tree was constructed using PhyML plugin in Geneious 6.1.8 suite (Biomatters Limited, Auckland, New Zeland) [26,27,28]. A substitution model was chosen using jModelTest software, v2.1.7, Department of Biochemistry, Genetics and Immunology, University of Vigo, Vigo, Spain) [29].

### 4.4. CYN Extraction and Preparation of Standard Curve

A medium made from a one-month-old culture of the *Anabaena lapponica* 966 strain (provided by J. Meriluoto from Åbo Akademi University) was filtered on Whatman GF/C filters, (diameter 47 mm) and conditioned on the combined SPE system (C18 cartridge connected to a PGC cartridge), as recommended by Metcalf and Codd [30]. CYN was eluted with 0.1% TFA in methanol, evaporated, and re-suspended in Mili-Q water (all reagents and materials from Sigma-Aldrich, St. Louis, MO, USA). Samples were stored at −20 °C and further purified by HPLC as described below, evaporated, and re-suspended in Mili-Q water. The concentration of purified CYN was determined spectrophotometrically, serial dilution (0.1–10 µg·mL^−1^) was analyzed by HPLC, and a calibration curve was prepared.

### 4.5. Cylindrospermopsin Purification and Analysis by HPLC-Diode Array UV Detection and MS/MS

Chromatographic separation was conducted on a Waters HPLC system consisting of a 600E multisolvent-delivery system, a 717plus autosampler, a 996 photodiode array detector (PDA), Millennium^32^ SS software and a Jetstream 2 plus column thermostat (Waters Corporation, Milford, MA, USA) using a Merck (Darmstadt, Germany) Purospher STAR RP-18e column (55 × 4 mm with 3 μm particles) protected by a 4 × 4 mm guard column, and a flow rate of 1 mL·min^−1^. The mobile phase consisted of a gradient of 0.05% aqueous TFA (solvent A) and 0.05% TFA in methanol (both from Sigma-Aldrich, St. Louis, MO, USA) (solvent B) with the following gradient program: 0% B for 7 min, ramped to 70% B in 0.1 min, held for 1.9 min, and then changed back to 0% B in 0.1 min and equilibrated for 8 min. The injection volume was 20 μL (biodegradation assays) and 150 μL (CYN purification). The retention time of CYN was 3.2 min. The PDA detector operated at 200–300 nm and the detection was conducted at 262 nm. The analyzed amounts of CYN were above the limit of detection (approx. 2 ng). Additionally, after purification, CYN was detected by Bruker HCT ultra ion-trap MS instrument (Bremen, Germany) operated in positive electrospray ion mode. The drying gas temperature and flow rate were set at 350 °C and 8.0 L·min^−1^, respectively. MS scans within the range *m*/*z* 100–500, and MS/MS scans of *m*/*z* 416.5 using an isolation width of 4.0 Da and fragmentation amplitude of 0.5 V with the SmartFrag function were performed.

### 4.6. Biodegradation Assays

In the first phase of the study, isolated bacterial strains were cultivated in peptone yeast extract medium (10 g of peptone, 5 g NaCl, 5 g of yeast extract in 1 L of distilled water, pH 7.2; Sigma-Aldrich, St. Louis, MO, USA) to rich stationary phase (2 days of incubation at 20 °C). The cells were washed twice in PBS buffer, centrifuged, and suspended in the same medium diluted 100 times with PBS buffer at pH 8 containing CYN (3 µg·L^−1^). The initial cell density in experimental samples was 10^5^ cells mL^−1^. CYN biodegradation assays were continued for six days, at 20 °C, in darkness. At time 0 and after 1, 4, 6, and 14 days, 300 µL of the sample was taken and centrifuged (12,000 g, 10 min, room temperature) in borosilicate glass chromatographic inserts and the reaction was stopped by freezing. The supernatant was analyzed to determine the residual CYN concentration by HPLC compared with a control (3 µg·L^−1^ CYN in 100× diluted medium). Based on this preliminary experiment, the R6 strain was selected for further investigation.

In the second phase of the study, all the experiments were carried out in darkness. To determine the impact of temperature and pH on the biodegradation of CYN by the R6 strain, two independent sets of two-week experiments were performed. Samples for CYN concentration analysis were taken at time 0 and after 1, 4, 6, and 14 days. Dependence of CYN biodegradation on temperature was tested, at 4, 20, and 30 °C at an initial pH of 8. The influence of pH (6.5, 8.0, 9.5) on the biodegradation was tested at 20 °C.

The growth of the R6 strain in the presence of CYN was monitored every two days based on the bacterial cell traditional plate counting. Bacterial cells were cultivated in medium diluted 100-fold to enhance the possible growth stimulation by CYN as one of the main carbon sources.

### 4.7. Statistical Analysis

ANOVA followed by Tukey’s test was applied to indicate statistically significant differences in the biodegradation of CYN in different temperatures and pH and in the growth of the R6 strain under different CYN concentrations or without CYN supplementation.

## Supplementary Materials

The following are available online at www.mdpi.com/2072-6651/8/3/55/s1. Table S1: Geographical position and limnological characteristics of the investigated lakes. Table S2: Strain identification: PCR procedure, BLAST search, and comparative data for phylogenetic analyzes. Table S3: Detailed presentation of statistic data that correspond to experiments described in Section 4.5. Figure S1: CYN biodegradation rates at different pH and temperature.

## Abbreviations

The following abbreviations are used in this manuscript:

BLAST: Basic Local Alignment Search Tool
CYN: cylindrospermopsin
MCs: microcystins
PCR: polymerase chain reaction
rDNA: ribosomal DNA (DNA coding ribosomal RNAs)

## References

[1] Antunes J.T., Leão P.N., Vasconcelos V.M. (2015). Cylindrospermopsis raciborskii: Review of the Distribution, Phylogeography, and Ecophysiology of a Global Invasive Species. Front. Microb..

[2] Kokociński M., Dziga D., Spoof L., Stefaniak K., Jurczak T., Mankiewicz-Boczek J., Meriluoto J. (2009). First report of the cyanobacterial toxin cylindrospermopsin in the shallow, eutrophic lakes of western Poland. Chemosphere.

[3] Kokocinski M., Meriluoto J., Spoof L., Gagala I., Jurczak T., Rejmonczyk E., Mankiewicz-Boczek J. (2011). Distribution and potential producers of cylindrospermopsin in western Poland. Eur. J. Phycol..

[4] Bogially S., Bruno M., Curini R., Di Corcia A., Fanali C., Laganà A. (2006). Monitoring algal toxins in lake water by liquid chromatography tandem mass spectrometry. Environ. Sci. Technol..

[5] Rücker J., Stüken A., Nixdorf B., Fastner J., Chorus I., Wiedner C. (2007). Concentrations of particulate and dissolved cylindrospermopsin in 21 *Aphanizomenon.*-dominated temperate lakes. Toxicon.

[6] Rzymski P., Poniedziałek B. (2014). In search of environmental role of cylindrospermopsin: A Review on Global Distribution and Ecology of its Producers. Wat. Res..

[7] Falconer I.R. (2005). Cyanobacterial Toxins in Drinking Water Supplies: Cylindrospermopsins and Microcystins.

[8] Kinnear S. (2010). Cylindrospermopsin: A Decade of Progress on Bioaccumulation Research. Mar. Drugs.

[9] Chiswell R.K., Shaw G.R., Eaglesham G.K., Smith M.J., Norris R.L., Seawright A.A., Moore M.R. (1999). Stability of cylindrospermopsin, the toxin from the cyanobacterium *Cylindrospermopsis. raciborskii*: Effect of pH, Temperature, and Sunlight on Decomposition. Environ. Toxicol..

[10] Humpage A.R., Falconer I.R. (2003). Oral toxicity of the cyanobacterial toxin cylindrospermopsin in male Swiss albino mice: Determination of no Observed Adverse Effect Level for Deriving a Drinking Water Guideline Value. Environ. Toxicol..

[11] Tsuji K., Masui H., Uemura H., Mori Y., Harada K.I. (2001). Analysis of microcystins in sediments using MMPB method. Toxicon.

[12] Klitzke S., Apelt S., Weiler C., Fastner J., Chorus I. (2010). Retention and degradation of the cyanobacterial toxin cylindrospermopsin in sediments - the role of sediment preconditioning and DOM composition. Toxicon.

[13] Klitzke S., Beusch C., Fastner J. (2011). Sorption of the cyanobacterial toxins cylindrospermopsin and anatoxin-a to sediments. Water Res..

[14] Senogles P., Smith M., Shaw G. Physical, Chemical and Biological Methods for the Degradation of the Cyanobacterial Toxin, Cylindrospermopsin. Proceedings of the Water Quality Technology Conference.

[15] Smith M.J., Shaw G.R., Eaglesham G.K., Ho L., Brookes J.D. (2008). Elucidating the factors influencing the biodegradation of cylindrospermopsin in drinking water sources. Environ. Toxicol..

[16] Wormer L., Cires A., Carrasco D., Quesada A. (2008). Cylindrospermopsin is not degradated by co-occuring natural bacterial communities during a 40-day study. Harmful Algae.

[17] Mohamed Z.A., Alamri S.A. (2012). Biodegradation of cylindrospermopsin toxin by microcystin- degrading bacteria isolated from cyanobacterial blooms. Toxicon.

[18] Nybom S.M., Salminen S.J., Meriluoto J.A. (2008). Specific strains of probiotic bacteria are efficient in removal of several different cyanobacterial toxins from solution. Toxicon.

[19] Gołdyn R., Podsiadłowski S., Kowalczewska-Madura K., Dondajewska R., Szeląg-Wasielewska E., Budzyńska A., Domek P., Romanowicz-Brzozowska W. (2010). Functioning of the Lake Rusałka ecosystem in Poznań (western Poland). Oceanol. Hydrobiol. Stud..

[20] Kokociński M., Soininen J. (2012). Environmental factors related to the occurrence of *Cylindrospermopsis. raciborskii* (Nostocales, Cyanophyta) at the north-eastern limit of its geographical range. Eur. J. Phycol..

[21] Kokociński M., Mankiewicz-Boczek J., Jurczak T., Spoof L., Meriluoto J., Rejmonczyk E., Hautala H., Vehniäinen M., Pawełczyk J., Soininen J. (2013). *Aphanizomenon. gracile* (Nostocales), a cylindrospermopsin -producing cyanobacterium in Polish lakes. Environ. Sci. Pollut. Res. Int..

[22] Janda J.M., Abbott S.L. (2007). 16S rRNA gene sequencing for bacterial identification in the diagnostic laboratory: Pluses, Perils, and Pitfalls. J. Clin. Microbiol..

[23] Mankiewicz-Boczek J., Gagala I., Jurczak T., Jaskulska A., Pawelczyk J., Dziadek J. (2015). Bacteria homologus to *Aeromonas.* capable of microcystin degradation. Open Life Sci..

[24] Dziga D., Wasylewski M., Wladyka B., Nybom S., Meriluoto J. (2013). Microbial degradation of microcystins. Chem. Res. Toxicol..

[25] Altschul S.F., Madden T.L., Schäffer A.A., Zhang J., Zhang Z., Miller W., Lipman D.J. (1997). Gapped BLAST and PSI-BLAST: A New Generation of Protein Database Search Programs. Nucleic Acids Res..

[26] Katoh K., Misawa K., Kuma K.I., Miyata T. (2002). MAFFT: A Novel Method for Rapid Multiple Sequence Alignment Based on Fast Fourier Transform. Nucleic Acids Res..

[27] Guindon S., Dufayard J.F., Lefort V., Anisimova M., Hordijk W., Gascuel O. (2010). New algorithms and methods to estimate maximum-likelihood phylogenies: Assessing the Performance of PhyML 3.0. Syst. Biol..

[28] Kearse M., Moir R., Wilson A., Stones-Havas S., Cheung M., Sturrock S., Buxton S., Cooper A., Markowitz S., Duran C. (2012). Geneious Basic: An Integrated and Extendable Desktop Software Platform for the Organization and Analysis of Sequence Data. Bioinformatics.

[29] Darriba D., Taboada G.L., Doallo R., Posada D. (2012). jModelTest 2: More Models, New Heuristics and Parallel Computing. Nat. Methods.

[30] Metcalf J., Codd G.A., Meriluoto J., Codd G.A. (2005). Solid Phase Extraction of Cylindrospermopsin in Filtered Water Samples. Cyanobacterial Monitoring and Cyanotoxin Analysis.

