# Supplementary material for: Cylindrospermopsin Biodegradation Abilities of Aeromonas sp. Isolated from Rusałka Lake
Source: Toxins (Basel). 2016 Feb 25;8(3):55. doi: 10.3390/toxins8030055 (PMC4810200; doi:10.3390/toxins8030055)
Supplement: Supplementary file 1 [file toxins-08-00055-s001.zip › toxins-111673-supplementary Table S1 Figure S1.pdf]

Supplementary Materials: Cylindrospermopsin Biodegradation Abilities of *Aeromonas* sp. Isolated from Rusałka Lake

Dariusz Dziga, Mikolaj Kokocinski, Anna Maksylewicz, Urszula Czaja-Prokop and Jakub Barylski

Table S1. Geographical position and limnological characteristics of the investigated lakes.

Geographical and Limnological Parameters	Lake Rusałka	Lake Kierskie Małe
Latitude	52°25′35″	52°29′12″
Longitude	16°52′40″	16°47′14″
Area (ha)	36.7	26.0
Average depth (m)	1.9	1.4
Max. Depth (m)	9.0	2.3
Secchi depth (m)	0.5	0.5
Chl-a (μg L^{-1})	60.1	108.2
CYN (μg L^{-1})	na	0.15

na: indicates not analysed.

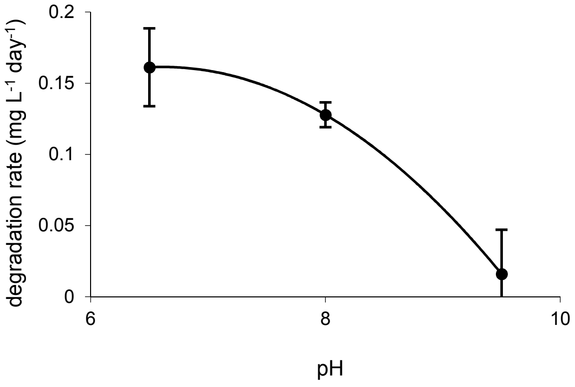

(**a**)

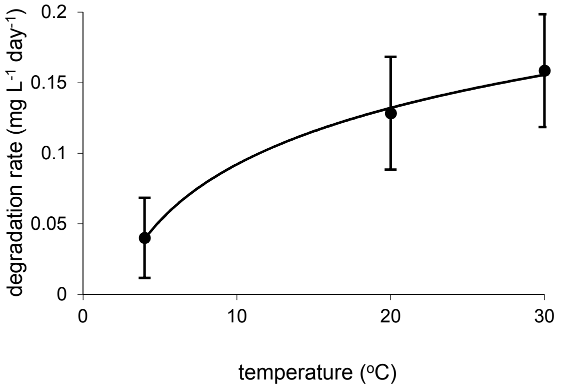

(**b**)

Figure S1. CYN biodegradation rates at different pH (**a**) and temperature (**b**). Errors indicate standard deviation ($n = 3$).
